# Complete Genome Sequence of *Nitrosomonas cryotolerans* ATCC 49181, a Phylogenetically Distinct Ammonia-Oxidizing Bacterium Isolated from Arctic Waters

**DOI:** 10.1128/genomeA.00011-17

**Published:** 2017-03-16

**Authors:** Marlen C. Rice, Jeanette M. Norton, Lisa Y. Stein, Jessica Kozlowski, Annette Bollmann, Martin G. Klotz, Luis Sayavedra-Soto, Nicole Shapiro, Lynne A. Goodwin, Marcel Huntemann, Alicia Clum, Manoj Pillay, Neha Varghese, Natalia Mikhailova, Krishna Palaniappan, Natalia Ivanova, Supratim Mukherjee, T. B. K. Reddy, Chew Yee Ngan, Chris Daum, Nikos Kyrpides, Tanja Woyke

**Affiliations:** a Utah State University, Logan, Utah, USA; b University of Alberta, Edmonton, Alberta, Canada; c Miami University, Oxford, Ohio, USA; d Queens College in The City University of New York, Flushing, New York, USA; e The Institute of Marine Microbes and Ecospheres, Xiamen University, Xiamen, China; f Oregon State University, Corvallis, Oregon, USA; g DOE Joint Genome Institute, Walnut Creek, California, USA; h Los Alamos National Laboratory, Bioscience Division, Los Alamos, New Mexico, USA

## Abstract

*Nitrosomonas cryotolerans* ATCC 49181 is a cold-tolerant marine ammonia-oxidizing bacterium isolated from seawater collected in the Gulf of Alaska. The high-quality complete genome contains a 2.87-Mbp chromosome and a 56.6-kbp plasmid. Chemolithoautotrophic modules encoding ammonia oxidation and CO_2_ fixation were identified.

## GENOME ANNOUNCEMENT

*Nitrosomonas cryotolerans* ATCC 49181 was isolated from surface seawater at 59°47′67″N, 151°55′08″W in 1980 and described in 1988 (1). *Nitrosomonas cryotolerans* is an obligate halophilic betaproteobacterium in the *Nitrosomonadaceae* (2) that is able to grow at temperatures as low as -5°C (3), the coldest temperature known for any bacterium in the *Nitrosomonadaceae*; *Nitrosomonas cryotolerans* was selected for sequencing based on phylogenetic and physiologic interests. Genome comparisons with *N. cryotolerans* will lead to better delineation of the *Nitrosomonas* genus.

*Nitrosomonas cryotolerans* was grown in marine medium containing 25 mM ammonium at 15°C (4). Cultures were harvested by centrifugation, and genomic DNA (gDNA) was isolated using the JGI cetyltrimethylammonium bromide (CTAB) protocol. The quality of gDNA was checked by gel electrophoresis and by amplification and sequencing of the V4 region of the small subunit (SSU) rRNA gene (5).

Genomic DNA was sequenced at the Department of Energy (DOE) Joint Genome Institute (JGI) using Pacific Biosciences (PacBio) technology (6). A PacBio SMRTbell library was constructed and sequenced on the PacBio RS platform, which generated 178,102 reads totaling 560.1 Mb. The input read coverage was 191.1×. Raw reads were assembled using HGAP version 2.2.0.p1 (7). The final assembly contains two contigs (one chromosome and one plasmid) totaling 2.87 Mbp in size. An earlier version accomplished using the Illumina HiSeq 2000 platform remained in 91 scaffolds, with nearly identical repeats of key gene clusters unresolved, and was not combined with the PacBio sequence for the complete genome reported here.

Genes were identified using Prodigal (8), using the DOE-JGI microbial annotation pipeline (9), followed by manual curation using GenePRIMP (10). The predicted coding sequences (CDSs) were translated and used to search the NCBI nonredundant, UniProt, TIGRFam, Pfam, KEGG, COG, and InterPro databases. Transfer RNA genes were identified using the tRNAscan-SE tool (11). Ribosomal RNA genes were found by searches against models from SILVA (12). Other noncoding RNAs were found using INFERNAL (13). Further gene prediction and manual curation were performed within the JGI Integrated Microbial Genomes platform (14, 15).

The genome of *N. cryotolerans* contains one ribosomal operon (SAMN02743940_1059 to SAMN02743940_1062). Metabolic modules encoding chemolithotrophic ammonia catabolism include three clusters each of ammonia-monooxygenase and hydroxylamine-oxidoreductase genes. The *amo* gene clusters have unique arrangements of neighboring genes, including the *amoCABEDcopCD* cluster (SAMN02743940_0955 to SAMN02743940_0961), *amoCABED* cluster (SAMN02743940_1533 to SAMN02743940_1537), and *amoCAB* cluster (SAMN02743940_2438 to SAMN02743940_2436). The genome contains 2 standalone *amoC* genes (SAMN02743940_2312 and 2350). Three *haoABcycAB* clusters are located at SAMN02743940_0346 to SAMN02743940_0349, SAMN02743940_0686 to SAMN02743940_0689, and SAMN02743940_1793 to SAMN02743940_1796. The *N. cryotolerans* genome contains a single gene cluster encoding the Calvin-Benson-Bassham cycle for carbon assimilation (SAMN02743940_1712 to SAMN02743940_1708 LysR, CbbL, CbbS, CbbQ, and CbbO) with highest nucleic acid sequence identity to homologous genes in *Nitrosomonas ureae* Nm10 (16) and *Nitrosomonas* sp. AL212 (17) related to the form 1A (green-like) subgroup. *Nitrosomonas cryotolerans* contains a cluster encoding urease (SAMN02743940_1408 to SAMN02743940_1413) and a urea transporter (SAMN02743940_1414). We identified an *nirK* gene at SAMN02743940_0821, putatively involved in nitrite processing, and a nitric oxide reductase *norCBQD* cluster at SAMN02743940_1672 to SAMN02743940_1675 suggestive of nitrifier denitrification. The nitrosocyanin protein, unique to ammonia-oxidizing bacteria (AOB), was encoded by SAMN02743940_2670. The evolutionary relationships in the *Nitrosomonadaceae* are currently under reconsideration.

### Accession number(s).

This complete genome sequence is deposited in ENA under accession numbers FSRO01000001 to FSRO01000002.
